# Sutureless Amniotic Membrane (ProKera®) and Intravenous Immunoglobulin in the Management of Ocular Complications of Stevens-Johnson Syndrome-Toxic Epidermal Necrolysis Overlap

**DOI:** 10.7759/cureus.16989

**Published:** 2021-08-08

**Authors:** Abdulhameed H Mahmood, Abdullah S Alharbi, Bader A Almanea, Anoud F Alsaati

**Affiliations:** 1 Ophthalmology, Salmaniya Medical Complex, Manama, BHR; 2 Ophthalmology, Anterior Segment Division, Prince Sultan Military Medical City, Riyadh, SAU; 3 Department of Family Medicine, Armed Forces Medical Center, Makkah, SAU; 4 Ophthalmology, Shaqra University, Riyadh, SAU

**Keywords:** stevens-johnson syndrome (sjs), toxic epidermal necrolysis (ten), cornea, ocular surface disease, prokera, intravenous immunoglobulin (ivig), dry eye disorder, symblepharon, ankyloblepharon, anterior segment

## Abstract

Purpose: To report a case of Stevens-Johnson syndrome-toxic epidermal necrolysis (SJS-TEN) overlap, with severe acute ocular manifestations successfully managed with sutureless Amniotic Membrane device ProKera^®^ (Bio-Tissue, Inc., Miami, FL) and topical steroids, followed by late complications that were successfully managed with intravenous immunoglobulin (IVIG) therapy.

Observations: A 24-year-old lady, known case of epilepsy, admitted to the burn unit with SJS-TEN overlap attributed to a recent change of her anti-convulsant therapy, with severe ocular manifestations, inability to open both eyes, and poor visual acuity. Early management included intensive topical steroids and lubrication, in addition to the application of a ProKera^®^ device. Despite achieving full epithelialization within two weeks with the improvement of ocular manifestations, the patient presented three weeks later with recurrence of conjunctival epithelial defects, partial ankyloblepharon, and severely dry corneas. These late sequelae were managed with bandage contact lens (BCL) application, intensive topical steroid, and lubrication in addition to IVIG therapy. After six cycles of IVIG therapy, ocular manifestations improved significantly and the patient achieved uncorrected visual acuity of 6/9 in both eyes.

Conclusion and importance: Existing evidence suggests that the use of IVIG in combination with systemic steroids in the early phase of SJS-TEN can reduce mortality, without affecting the final visual outcome in patients with ocular manifestations. This case highlights the possible role of IVIG therapy alone - without systemic steroids - in managing and preventing long-term ocular complications of SJS-TEN.

## Introduction

Stevens-Johnson syndrome (SJS) and toxic epidermal necrolysis (TEN) are rare, severe, blistering illnesses of skin and mucous membranes. Both diseases are caused by a delayed-type hypersensitivity incited by the use of drugs, most commonly anticonvulsants, antibiotics, nonsteroidal anti-inflammatory drugs, and allopurinol [1,2]. They both generate clinical features similar to a partial-thickness skin burn image, but TEN involves a more extensive body surface compared to SJS. In SJS, less than 10% of body surface area is affected, SJS/TEN overlap affects up to 30% of body surface area, while in TEN greater than 30% of the body surface area is involved ‎[2].

SJS has an incidence of approximately 6 cases per 1,000,000 with a 3% mortality rate whereas TEN has an incidence of approximately 1 to 2 cases per 1,000,000 annually with a mortality rate of 25% to 40% ‎[3].

Ocular manifestations are common, occurring in 50% to 88% of patients with SJS/TEN, and they include membranous conjunctivitis, symblepharon formation, and epithelial defects of the corneal epithelium, conjunctival epithelium, and lid margins in the acute phase. Chronic ocular consequences, varying from dry eyes to ocular surface keratinization, meibomian glands dysfunction, and limbal stem cell deficiency can also occur. In addition to fluid replacement and supportive care in the acute phase of the disease, the current standard of care for the management of ocular complications includes aggressive topical steroid, lubrication, fornix sweeping, and amniotic membrane transplant (AMT) to address ocular surface de-epithelialization [3,4].

Current evidence suggests that systemic cyclosporine or the combination of corticosteroids and intravenous immunoglobulin (IVIG) in the acute phase could reduce mortality in selected patients [5]. However, until now, no evidence exists on the therapeutic benefits of systemic immunomodulatory therapy on the prevention of chronic ocular complications of SJS/TEN or the final visual outcome in such patients [4,5].

The utilization of the amniotic membrane, the placenta's innermost layer, has a proven anti-inflammatory and anti-fibrotic benefits in many inflammatory conditions of the ocular surface, such as SJS/TEN and ocular burns. Therefore, early AMT remains the single most important surgical intervention in patients with SJS/TEN with moderate to severe ocular manifestations ‎[6,7].

Several case reports and series have previously shown that AMT can suppress inflammation, avoid ocular surface ulceration, and aid healing in the acute phase of SJS/TEN ‎[1,8-11].

The traditional sutured AMT, covering the entire cornea, bulbar and palpebral conjunctival surfaces and the lid margins, is a time-consuming and onerous procedure that is not always possible in view of the systemic manifestations of SJS/TEN, which can render the patient unfit for a long OR-based surgical procedure under local or general anesthesia ‎[12].

Therefore, several sutureless fixation methods were adopted by many ophthalmologists to allow for easier bedside or clinic-based application of AMT. Some of those techniques rely on fixating the tissue on a ring-like device made from different materials such as intravenous, and feeding tubes, which simultaneously act as a symblepharon ring ‎[13,14]. Others utilize cyanoacrylate glue to fixate the tissue as an alternative sutureless method for AMT [15].

The sutureless, adhesiveless AMT device ProKera® (Bio-Tissue, Inc., Miami, FL) has recently emerged as a quick, safe, and effective method for bedside or clinic-based application of amniotic membrane tissue in the treatment of various ocular surface diseases [16,17].

ProKera® application has already shown encouraging outcomes in terms of suppressing inflammation, healing and preventing further corneal epithelial defects, and preserving visual acuity without significant cicatricial complications or limbal stem cell deficiency ‎[1,3,17-20].

## Case presentation

A 24-year-old female, who is a known case of epilepsy, was admitted to the burn unit under dermatology and internal medicine care, with severe dehydration and painful erythematous macular rash involving around 25-30% of her body including the face, all of which started 10 days after switching her anti-convulsant therapy to Keppra (Levetiracetam). The patient was diagnosed with SJS-TEN overlap and was treated with intravenous (IV) methylprednisolone 500 mg once per day for the first two days, followed by oral dexamethasone 0.5 mg four times daily from day 3 until day 5, in addition to systemic cyclosporine A at a dose of 150 mg per day (equivalent to 3.75 mg/kg/day), which continued until day 10. The patient also received subcutaneous (SC) etanercept 50 mg on day 2 of admission.

Ophthalmology consultation was requested to address her severe bilateral eye pain, inability to open the eyes, and poor visual acuity. On examination, there was severe upper and lower eyelid edema with epidermal sloughing, severe congestion, chemosis, and hemorrhagic conjunctival membranes in both eyes. Examination using portable slit-lamp and fluorescein staining also showed total corneal and conjunctival epithelial defects affecting the bulbar and palpebral conjunctiva in the right eye, and 50% corneal epithelial defect with palpebral conjunctival epithelial defects in the left eye. Visual acuity could not be assessed as the patient was unable to open her eyes.

The patient was unfit for local or general anaesthesia to undergo a full ocular surface AMT, thus, the decision was made to apply a ProKera® device to both eyes, after thorough irrigation and removal of conjunctival membranes.

Intensive one-hourly preservative-free lubricating eye drops (sodium hyaluronate 0.2%) were also initiated, in addition to prednisolone acetate 1% drops four times daily, moxifloxacin 0.5% drops four times daily, combined with daily sweeping of the fornices and lid margins to prevent symblepharon and/or ankyloblepharon formation. 

**Figure 1:**
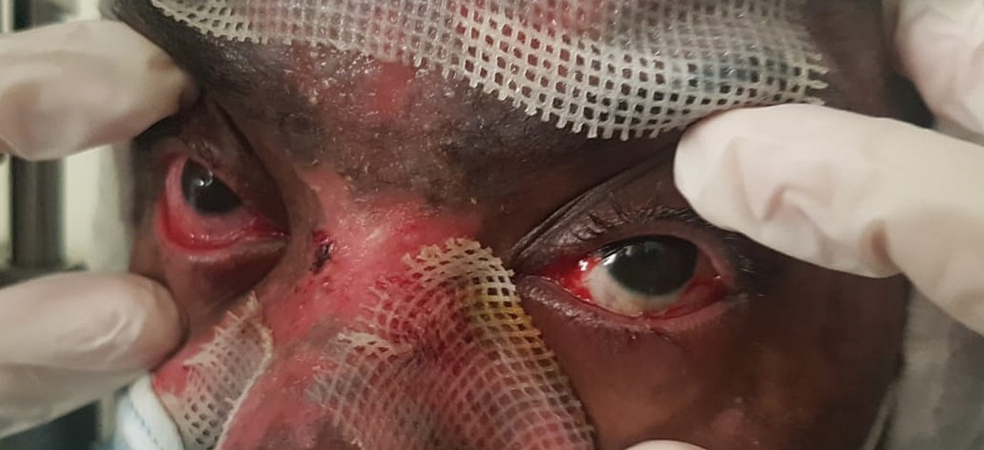
External photo of both eyes, showing significant improvement of congestion in the left eye. The right eye is still severely congested.

**Figure 2:**
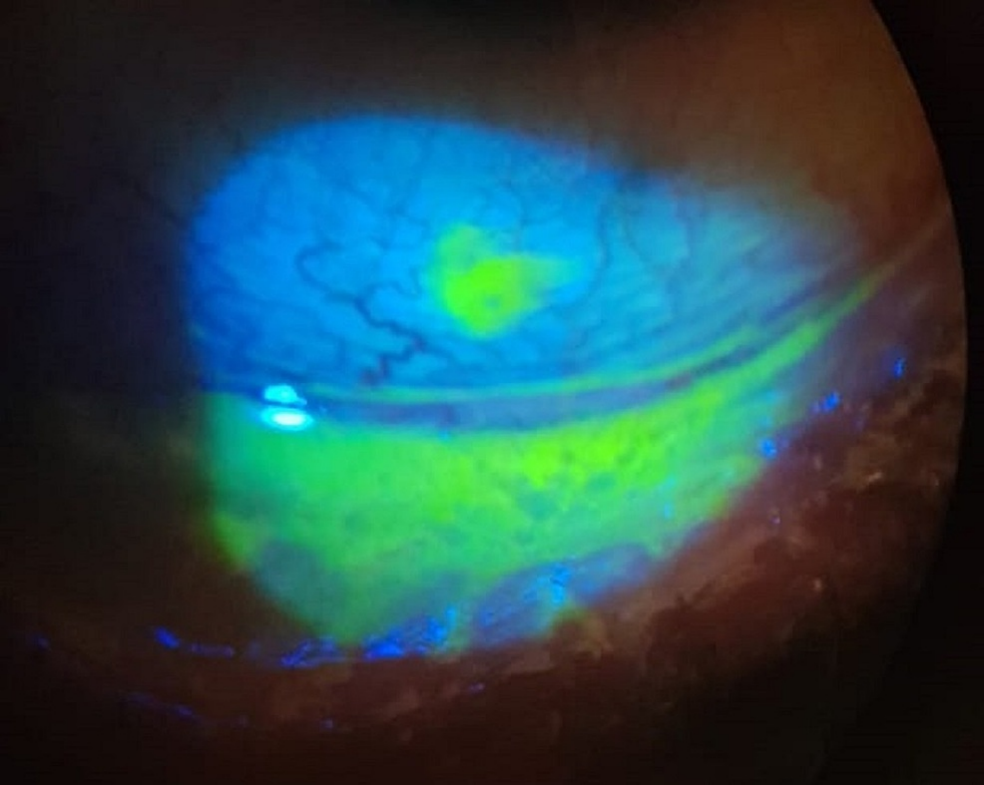
Slit-lamp photo of the right eye on day 10, showing inferior bulbar and palpebral conjunctival epithelial defects.

**Figure 3:**
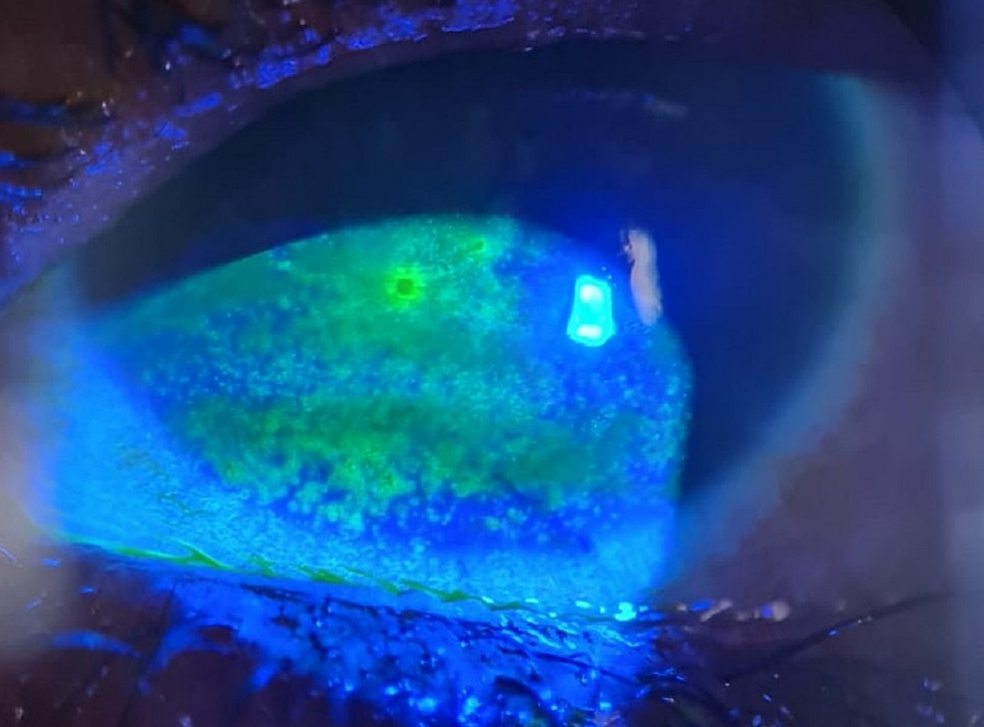
Slit-lamp photo of the left eye on day 10, showing small central CED with diffuse SPKs. CED: corneal epithelial defect, SPK: superficial punctate keratopathy.

On day 10, both ProKera® devices were removed to reassess the ocular surface. External examination showed significant improvement with much less congestion in the left eye (Figure 1). Slit-lamp examination revealed that the right eye still has 30% corneal epithelial defect with small areas of conjunctival epithelial defects inferiorly (Figure 2), and the ProKera® device was reapplied. The left eye showed complete conjunctival re-epithelialization with small corneal epithelial defect and diffuse multiple superficial punctate keratopathies (SPKs) (Figure 3). A bandage contact lens (BCL) was applied to this eye for the patient’s comfort. The patient also reported improvement of symptoms and visual acuity at the time of examination was 6/120 in the right eye, and 6/36 in the left eye.

On day 14, the patient’s systemic condition was stable, with complete skin re-epithelialization. Ophthalmic examination showed a visual acuity of 6/60 in the right eye and 6/36 in the left eye. Complete corneal and conjunctival re-epithelialization was also achieved in both eyes with diffuse SPKs, and the patient was discharged on BCL in both eyes, intensive lubrication, prednisolone acetate 1% four times daily (to be tapered weekly), cyclosporine-A 0.05% four times daily, and prophylactic moxifloxacin 0.5% twice daily.

The patient was followed up weekly, and her symptoms were improving gradually. Uncorrected visual acuity improved to 6/12 in both eyes, with minimal congestion and few SPKs. BCLs were discontinued uneventfully after two weeks (day 28) and prednisolone acetate 1% was switched to fluorometholone 0.1% (three times daily initially with weekly tapering) and lubricating ointment was also added to her regimen.

**Figure 4:**
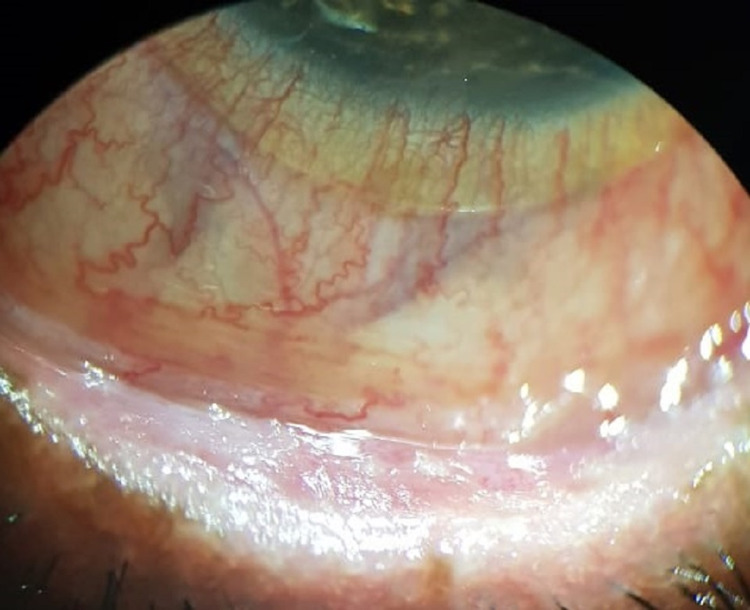
Slit-lamp photo of the left eye, with everted lower eyelid, showing lid margin keratinization with loss of grey line and posteriorly migrated MCJ. MCJ: mucocutaneous junction.

**Figure 5:**
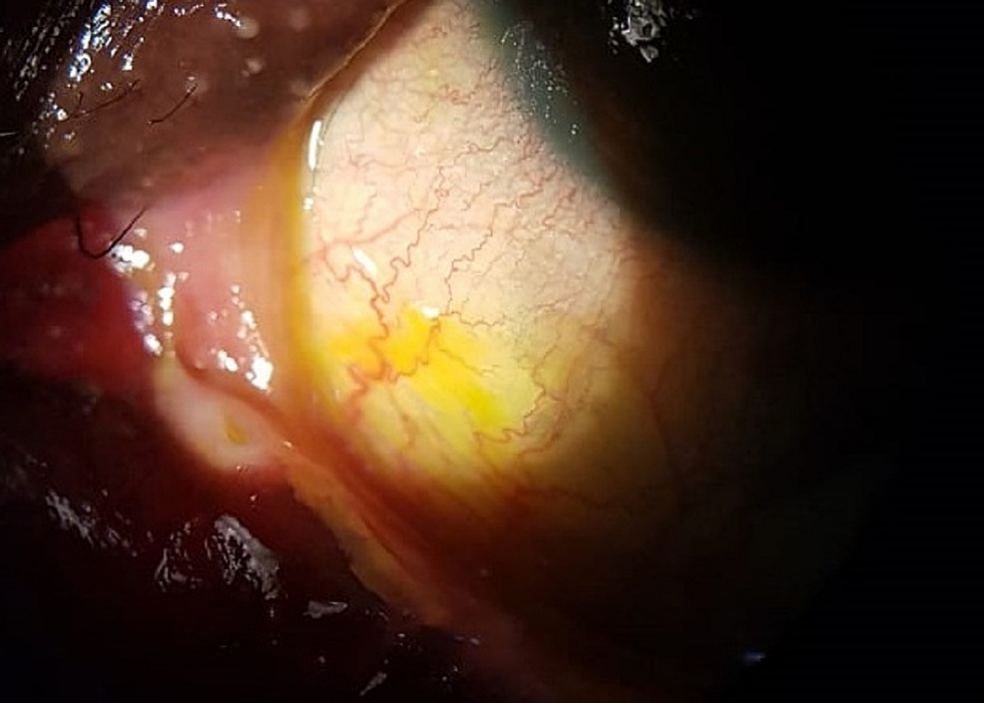
Slit-lamp photo of the left eye showing nasal partial ankyloblepharon, obliterating the upper lacrimal punctum, while the lower punctum remains patent.

**Figure 6:**
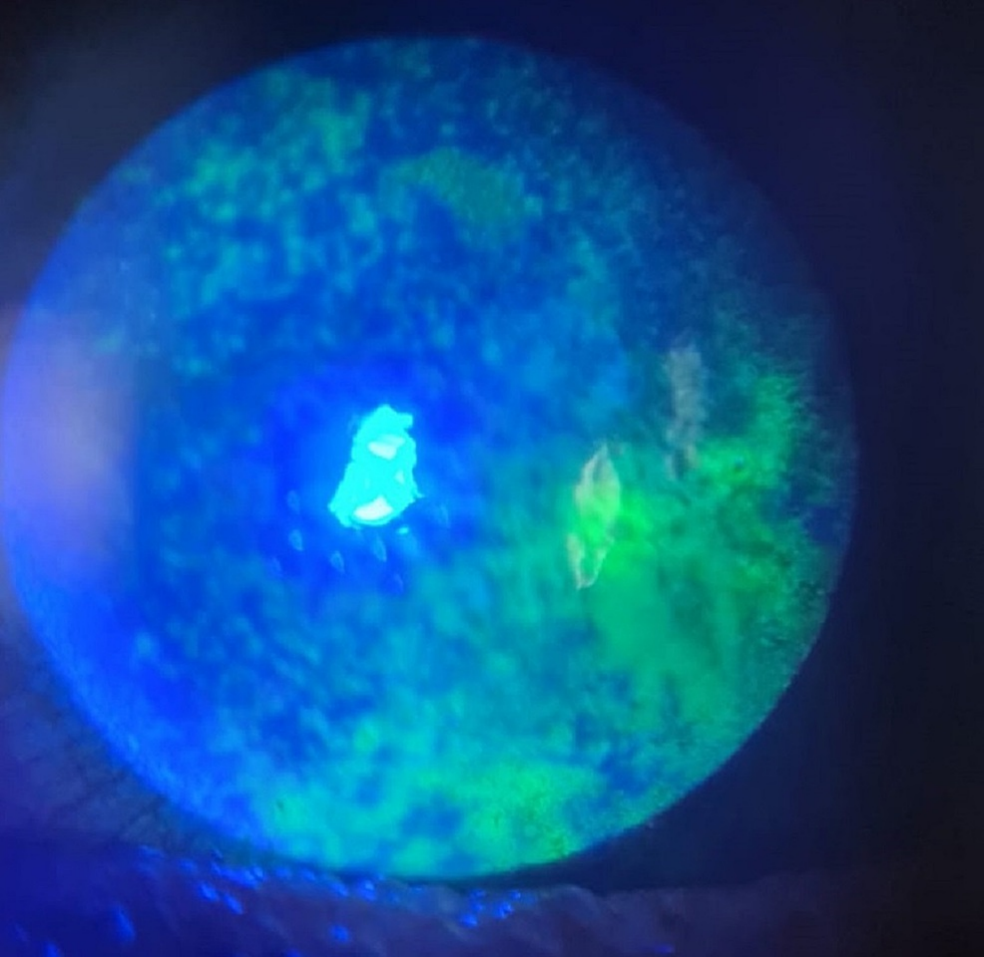
Slit-lamp photo of the right eye showing confluent SPKs. SPK: superficial punctate keratopathy.

However, the patient presented three weeks later with worsening of her symptoms and decreased visual acuity to 6/18 in the right eye and 6/24 in the left eye. Examination revealed early lid margin keratinization, partial ankyloblepharon, and diffuse SPKs in both eyes (Figures 4-6). Adhesions were separated, and the patient was restarted on prednisolone acetate 1% eye drops in addition to Maxitrol ointment (Neomycin, Polymyxin B, and Dexamethasone 0.1%). BCLs were also applied to both eyes.

After discussing systemic treatment options with the patient, the decision was made to start intravenous immunoglobulin (IVIG) therapy for four cycles over four months (2 g/kg/cycle given over two days), with the possibility to extend treatment to six cycles if the favorable response is achieved.

The patient noticed an improvement in her symptoms after the second cycle, with significant improvement of ocular surface wetness, and visual acuity of 6/12 in the right eye and 6/18 in the left eye, and BCLs were discontinued. Both Maxitrol ointment and prednisolone acetate drops were also discontinued after two cycles of IVIG, and the patient was kept on lubricating eye drops (sodium hyaluronate 0.2%) and ointments, fluorometholone 0.1% eye drops with slow tapering and cyclosporine 0.05% for the remainder of the treatment plan. IVIG therapy was continued for a total of six cycles.

**Figure 7:**
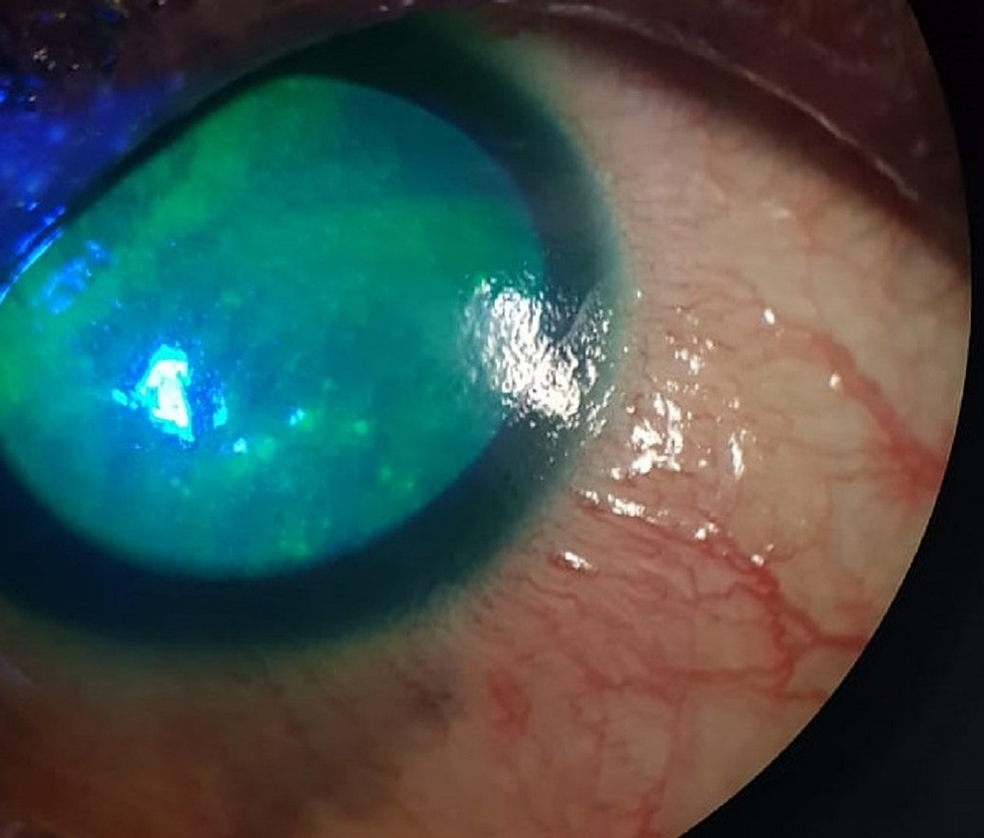
Slit-lamp photo of the right eye after six cycles of IVIG therapy, showing only SPKs in a quiet eye. SPK: superficial punctate keratopathy, IVIG: intravenous immunoglobulin.

**Figure 8:**
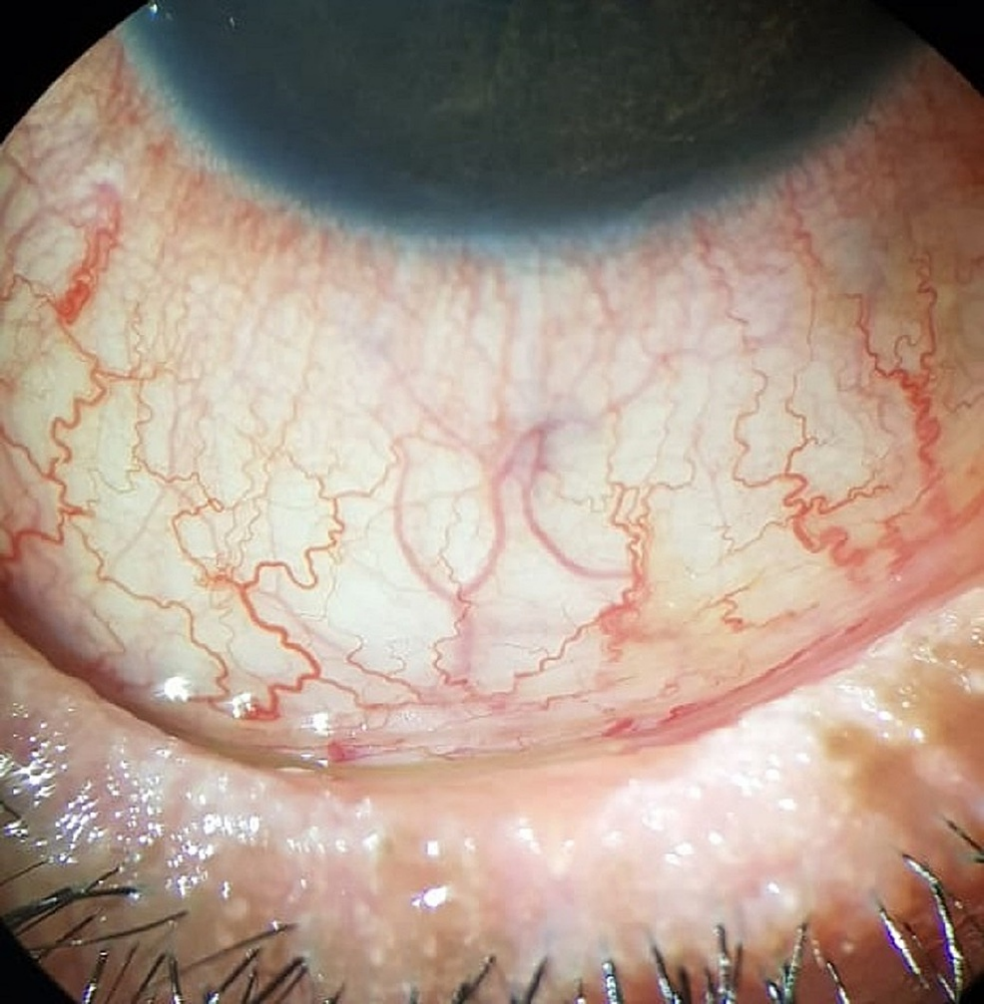
Slit-lamp photo of the left eye after six cycles of IVIG therapy, showing a quiet eye, improved lid margin irregularity, with keratinization still present. IVIG: intravenous immunoglobulin.

After six cycles, the patient’s symptoms further improved and on her last visit to the clinic, she achieved visual acuity of 6/9 in both eyes. Her examination showed quiet eyes and a wet ocular surface with only a few SPKs (Figure 7). No new symblepharon or ankyloblepharon formation was noted. The lid margin anatomy and irregularity improved significantly. However, signs of meibomian gland dysfunction and keratinization are still present (Figure 8). The patient still has symptoms of dry eyes that are currently being managed sufficiently with lubricating eye drops and ointments.

## Discussion

Ocular involvement is common in SJS and TEN and can be severe and blinding. The goal of treatment in these patients, besides stabilizing the patient’s general condition, is to prevent late ocular cicatricial complications, by aggressively addressing the inflammation, membranous conjunctivitis, and epithelial sloughing in the acute phase, which is usually achieved with AMT, aggressive lubrication, and topical steroid therapy [4,7,8]. In our case, the patient’s general condition was not stable enough to go for a traditional sutured AMT. The ProKera® device worked well as an alternative method in hastening corneal and conjunctival epithelial healing, preventing limbal stem-cell deficiency, in addition to its anti-inflammatory effect. However, it is important to note while using this device that the polycarbonate ring of the device does not necessarily reach deep into the fornices. Therefore, daily sweeping with a wet cotton tip applicator is still necessary to break new symblephara, preventing shortening of the fornices, and also preventing adhesion of the denuded tarsal conjunctiva to the polycarbonate ring of the ProKera® device itself, which could delay healing.

The rarety of SJS and TEN limits the ability of scientists to study and directly compare therapeutic agents, and even more so, comparing their effects on ocular outcomes particularly. The most commonly used systemic immunomodulatory agents in the treatment of SJS and TEN are corticosteroids, immunoglobulin, and cyclosporine. While there is strong evidence suggesting that the use of such agents is beneficial in reducing both mortality and recovery time in patients with SJS and TEN, their role in preventing ocular complications or improving visual outcomes remains questionable at best [5,21,22].

In a retrospective study of 28 patients in 2005, Yip et al. [23] found no difference in terms of frequency and severity of ocular complications between patients who received high-dose IVIG therapy and those who did not. On the other hand, a Korean retrospective observational case series of 51 patients, found that younger patients with SJS or TEN tend to have poorer ocular outcomes than older patients and that early treatment with steroid or immunoglobulin therapy could improve these outcomes [24]. The latter study, in addition to the patient’s young age, her hesitance to take oral steroids, and the fact that she experienced deterioration of renal function while taking systemic cyclosporine during her hospital stay, all encouraged us to start the patient on IVIG therapy. Especially, that we strongly felt the need to add a systemic agent to her topical regimen, in the hope to suppress the ocular inflammation in a timely manner, thus preventing chronic cicatricial sequelae.

The response seen in the 24-year-old patient presented in our case report after only two cycles of IVIG therapy is indeed encouraging; however, further and larger studies are needed to produce stronger evidence to support the use of such an expensive therapy more routinely. Future studies should also focus on the best timing, duration, and number of cycles needed to give the best results in terms of ocular outcomes.

## Conclusions

Ocular complications in patients with SJS and TEN are common. Sutureless and adhesiveless ProKera® device can be used in the acute phase as an efficient, safe, and less invasive alternative to the traditional sutured AMT and should be considered for selected patients. Close follow-up for these patients is crucial as inflammation and corneal or conjunctival erosions could recur even after complete re-epithelialization. IVIG therapy combined with topical therapy might have a role in improving ocular outcomes in patients with SJS and TEN. However, further and larger studies are still needed to confirm its efficacy and determine the best timing and duration of treatment.
